# Methods to test for association between a disease and a multi-allelic marker applied to a candidate region

**DOI:** 10.1186/1471-2156-6-S1-S101

**Published:** 2005-12-30

**Authors:** Rachid El Galta, Li Hsu, Jeanine J Houwing-Duistermaat

**Affiliations:** 1 Department of Medical Statistics and Bioinformatics, Leiden University Medical Centre, Leiden, P.O. Box 9604, 2300RC Leiden, The Netherlands; 2 Modeling and Methods, Biostatistics Program, Fred Hutchinson Cancer Research Center, Seattle, WA, USA

## Abstract

We report the analysis results of the Genetic Analysis Workshop 14 simulated microsatellite marker dataset, using replicate 50 from the Danacaa population. We applied several methods for association analysis of multi-allelic markers to case-control data to study the association between Kofendrerd Personality Disorder and multi-allelic markers in a candidate region previously identified by the linkage analysis. Evidence for association was found for marker D03S0127 (*p *< 0.01). The analyses were done without any prior knowledge of the answers.

## Background

Terwilliger [1] proposed a powerful method for the association analysis between a disease and a multi-allelic marker. The model assumes that only one marker allele is associated with the disease and that any marker allele may be associated with the disease with prior probability equal to its allele frequency in the population. The excess allele in the cases is modelled by a parameter λ, the population attributable risk [2]. The likelihood of the data given the allele frequencies and the parameter λ is the weighted sum of the conditional likelihood functions given that an allele is associated with the disease over all marker alleles with weights equal to the allele frequencies. Hence, more weight is assigned to more frequent marker alleles.

To test the null hypothesis (λ = 0) against the alternative hypothesis (λ > 0), Terwilliger [1] proposed a likelihood ratio (LR) statistic. However, this statistic appeared to be conservative and computation of the maximum-likelihood estimates might be slow. Another point mentioned by Sham et al. [3] is that this LR test statistic might not be robust against model deviation, especially when there is more than one allele associated with the disease. With this consideration, we derived the corresponding score statistic U, which is a linear combination of Pearson's χ^2 ^and a weighted sum of observed minus expected allele counts in cases. The score test is locally most powerful and because it is evaluated under the null hypothesis, it is expected to be robust against model deviation [4]. The score statistic U is easy to compute, which enables one to use Monte-Carlo permutations to estimate the empirical *p*-value of the test statistic [5]. For a large number of alleles Pearson's χ^2 ^follows asymptotically a normal distribution [6]. Hence, for a large sample size and for a large number of marker alleles the distribution of the score test U under the null hypothesis can be approximated by a normal distribution. Another alternative may be to replace the weights in the LR statistic proposed by Terwilliger by equal weights, which might be suitable if the associated allele is less common.

For replicate 50 of the Danacaa population we applied the Pearson's χ^2^, the score test, and Terwilliger's LR test to microsatellite markers D03S0124, D03S0125, D03S0126, and D03S0127 to test their association with Kofendrerd Personality Disorder (KPD). The allelic distribution was compared between a sample of 100 cases and a sample of 50 controls. In order to ensure high power, one might either select more controls, because they are easier to ascertain than cases or to compare the allele frequencies in cases to the allele frequencies in the population if they are known. Because the allele frequencies in controls were supplied by the Genetic Analysis Workshop 14 (GAW14), we considered the latter option to verify the result of markers that showed significant association with KPD.

## Materials and methods

### Score test

Suppose we have a multi-allelic marker. Let p_i _be the frequency of the *i*^th ^allele in the controls. Suppose we have n_1 _unrelated case chromosomes and n_2 _unrelated control chromosomes. Let x_i _and y_i _be the *i*^th ^allele counts in cases and controls respectively. The score statistic corresponding to the likelihood proposed by Terwilliger [1] is

U = ∑(x_i _- n_1_p_i_)^2^/p_i _- ∑(x_i _- n_1_p_i_)/p_i_,

where the sum is taken over the alleles. When the allele frequencies are unknown, p_i _can be estimated by the frequencies in combined sample (x_i_+y_i_)/(n_1_+n_2_). When more than one allele is associated with the disease, the score test U is expected to perform better than the LR, because it sums over the contributions of the alleles.

### Data analysis

Firstly we selected four replicates from each of the Aipotu, Karangar, and Danacaa populations to perform genome-wide linkage analysis, i.e., we analyzed 12 replicates. Each replicate consisted of 100 nuclear families. For each replicate we applied the single-point S_pairs _allele-sharing scoring function [7] as implemented in the MERLIN program [8] to search for regions with evidence for linkage. The parental genotypes were used to compute the probabilities of sharing 0, 1, or 2 alleles identically by descent. A region on chromosome 3 showed a significant linkage to latent disease locus for several populations at level 0.0001.

For testing the association using the proposed methods, we selected replicate 50 of the Danacaa population, because in this replicate marker D03S0127 showed highly significant linkage to the disease locus with a LOD score greater than 6 (*p *< 0.0001). Flanking markers D03S0126, D03S0125, and D03S0124 showed borderline linkage with LOD scores equal to 1.35, 1.45, and 2.42, respectively.

In order to obtain marker genotypes for 50 unrelated controls for the association analysis we purchased packets 149 to 153. The first affected in each family (*n *= 100) was used as a case regardless of being child or parent. We tested for the Hardy-Weinberg equilibrium to each microsatellite marker in the controls. Then we applied the score test U, Pearson's χ^2^, and Terwilliger's LR to study association with KPD. For the score test U and Pearson's χ^2^, we used Monte-Carlo permutations to estimate the empirical *p*-values. *p*-Values lower than 0.05 were considered to be significant.

As an alternative to using the controls, we also used the provided allele frequencies as reference allelic frequency distribution for Pearson's χ^2^, the score U, and Terwilliger's LR. We also used Terwilliger's LR with equal weights.

Finally, additional SNPs in the vicinity of the associated marker D03S0127 were tested for association and the linkage disequilibrium between markers was studied in this region.

## Results

All markers were in Hardy-Weinberg equilibrium proportions. Table 1 presents the *p*-values for the association analysis of various markers with the disease. Marker D03S0127 appeared to be highly significantly associated with the disease. The score U and Pearson's χ^2 ^gave about the same *p*-value (*p *= 0.008, 0.007), whereas Terwilliger's LR yielded somewhat a larger *p*-value (*p *= 0.033). For this marker, allele 1 and 3 were present 2 and 3.3 times more often in cases than in controls respectively, whereas allele 6 occurred approximately 2.8 times as often in controls as in cases. Marker D03S0125 showed borderline significant association with KPD. Next we repeated the analysis of association between KPD and marker D03S0127 using the provided allele frequencies of 0.070, 0.206, 0.100, 0.114, 0.048, 0.111, 0.154, and 0.197 for allele 1 to 8, respectively. Again the score statistic U and Pearson's χ^2 ^yielded similar empirical *p*-values (*p *= 0.027), while LR of Terwilliger and LR with equal weights gave an asymptotic *p*-value of 0.029 and 0.023, respectively. Compared to the given allele frequencies only allele 3 showed some excessive frequency in the cases, and it occurred about 1.7 times as often in cases as in the population.

## Discussion

In this paper we reported results of several methods for studying association between a disease and a multi-allelic marker. Marker D03S0127 located at chromosome 3 showed significant association with the disease. Both score U and Pearson's χ^2 ^tests gave somewhat lower *p*-values than the Terwilliger's LR test. Further examination shows that marker D03S0127 appeared to have two positively associated alleles. When we assumed known allele frequencies, only one allele was positively associated with the disease and all test statistics yielded similar *p*-values. Perhaps the fact that there are two associated alleles might be the reason that Terwilliger's LR test yielded somewhat larger *p*-value in this dataset. To study whether this holds in general, an extensive simulation study is needed.

In addition to Pearson's χ^2 ^and LR, a new test statistic was applied to the GAW14 simulated data. The new test statistic is derived based on the score function under the null hypothesis. So it possesses the usual optimal properties of other score test statistics: locally most powerful and robust against model misspecification. In contrast to the LR test statistic, the new score statistic is very easy to compute and uses Monte-Carlo methods to derive empirical *p*-values. Details of the derivation of this score statistic as well as a simulation study of its power will be extensively provided in another paper.

The parameter λ is a preferred measure of allelic association because it is directly related to recombination fraction and it is less sensitive to allele frequencies than other measures [2]. However, when allelic association is modelled by means of λ, it is not straightforward to adjust for other covariates. Houwing-Duistermaat and Elston [9] discussed various ways to quantify allelic association and estimate the location of a gene responsible for disease using logistic regression models. As an alternative to λ, the log relative risk as measured by the regression coefficient in the logistic model may be used to allow for adjustment of other covariates. More research is needed to build this kind of flexible model.

Applying Pearson's χ^2 ^with one degree of freedom to 19 SNPs revealed strong association between KPD disease and two di-allelic markers in this region: SNP B03T3056 and SNP B03T3057. Furthermore, LD observed between B03T3056 and B03T3057 and B03T3056 and D03S0127 further confirms the precedent results.

## Conclusion

All test statistics showed significant association between D03S0127 and KPD. Probably due to the presence of more than one positively associated allele, the Pearson's χ^2 ^and score tests yielded lower *p*-values than the Terwilliger's LR test in this dataset.

## Abbreviations

GAW14: Genetic Analysis Workshop 14

KPD: Kofendrerd Personality Disorder

LR: Likelihood ratio

## Authors' contributions

REG participated in method development, prepared data, carried out data analysis, participated in interpreting results, and drafted the manuscript. LH participated in method development and in interpreting results. JJH-D participated in method development and in interpreting results, and supervised the drafting of the manuscript.

## Figures and Tables

**Table 1:** Results of association tests for multi-allelic markers

Marker	Number of alleles	Allele count (frequency)		Score U*	Terwilliger's LR	Pearson's χ^2^*
					
		Cases	Controls			
D03S0124						
	5	09 (0.045)	06 (0.060)	0.910	0.500	0.930
		31 (0.155)	16 (0.160)			
		28 (0.140)	11 (0.110)			
		75 (0.375)	39 (0.390)			
		57 (0.275)	28 (0.280)			
						
D03S0125						
	4	12 (0.060)	03 (0.030)	0.069	0.070	0.053
		79 (0.395)	27 (0.270)			
		55 (0.275)	31 (0.310)			
		54 (0.270)	39 (0.390)			
						
D03S0126						
	7	22 (0.110)	16 (0.160)	0.426	0.500	0.428
		08 (0.040)	05 (0.050)			
		64 (0.320)	37 (0.370)			
		16 (0.080)	10 (0.10)			
		23 (0.115)	06 (0.060)			
		51 (0.255)	18 (0.180)			
		16 (0.080)	08 (0.080)			
						
D03S0127						
	8	12 (0.060)	03 (0.030)	0.008	0.033	0.007
		33 (0.165)	20 (0.200)			
		33 (0.165)	05 (0.050)			
		23 (0.115)	13 (0.130)			
		08 (0.040)	04 (0.040)			
		14 (0.070)	20 (0.200)			
		39 (0.195)	16 (0.160)			
		38 (0.190)	19 (0.190)			

*p-Values were obtained using 10000 Monte-Carlo simulations
